# Choline supplementation regulates gut microbiome diversity, gut epithelial activity, and the cytokine gene expression in gilts

**DOI:** 10.3389/fnut.2023.1101519

**Published:** 2023-02-02

**Authors:** Xiaoshu Zhan, Lauren Fletcher, David Huyben, Haiming Cai, Serena Dingle, Nanshan Qi, Lee-Anne Huber, Bingyun Wang, Julang Li

**Affiliations:** ^1^Department of Life Science and Engineering, Foshan University, Foshan, Guangdong, China; ^2^Department of Animal Biosciences, University of Guelph, Guelph, ON, Canada; ^3^Institute of Animal Health, Guangdong Academy of Agricultural Sciences, Guangzhou, Guangdong, China

**Keywords:** gut microbiome, choline supplement, ovarian development, gilts, intestinal epithelial activity

## Abstract

Choline is an essential nutrient that is necessary for both fetal development and maintenance of neural function, while its effect on female ovarian development is largely unexplored. Our previous study demonstrated that choline supplementation promotes ovarian follicular development and ovulation, although its underlying mechanism was unclear. To uncover the potential regulation pathway, eighteen female Yorkshire × Landrace gilts were fed with either standard commercial diet (Control group, *n* = 9) or choline supplemented diet (Choline group, additional 500 mg/kg of control diet, *n* = 9) from day 90 of age to day 186. At day 186, feces samples were analyzed for effects on the gut microbiome using 16S ribosomal RNA gene V3–V4 region sequencing with Illumina MiSeq, serum samples were analyzed for trimethylamine (TMA) and trimethylamine-N-oxide (TMAO) using HILIC method, and jejunum tissues were analyzed for immune related gene expression using qRT-PCR. Our results show that choline supplementation did not alter the circulating level of TMA and TMAO (*P* > 0.05), but rather increased gut microbiome alpha diversity (*P* < 0.05). Beta diversity analysis results showed that the choline diet mainly increased the abundance of *Firmicutes*, *Proteobacteria*, and *Actinobacteria*, but decreased the abundance of *Bacteroidetes*, *Spirochaetes*, and *Euryarchaeota* at the phyla level. Meta-genomic analysis revealed that choline supplementation activated pathways in the gut microbiota associated with steroid hormone biosynthesis and degradation of infertility-causing environmental pollutants (bisphenol, xylene, and dioxins). To further verify the effect of choline on intestinal activity, a porcine intestine cell line (IPEC-J2) was treated with serial concentrations of choline chloride *in vitro*. Our data demonstrated that choline promoted the proliferation of IPEC-J2 while inhibiting the apoptotic activity. qRT-PCR results showed that choline significantly increased the expression level of *Bcl2* in both IPEC-J2 cells and jejunum tissues. The expression of *IL-22*, a cytokine that has been shown to impact ovarian function, was increased by choline treatment *in vitro*. Our findings reveal the beneficial effect of choline supplementation on enhancing the gut microbiome composition and intestinal epithelial activity, and offer insights into how these changes may have contributed to the ovarian development-promoting effect we reported in our previous study.

## 1. Introduction

Choline is an essential nutrient for both humans and animals, serving as precursor for phosphatidylcholine and acetylcholine to maintain cellular membrane structure and neural function. Choline supplementation is an important nutritional strategy for pregnant females to improve the health of their fetus (1). It is reported that the concentration of choline in the maternal plasma during the third trimester of pregnancy is positively associated body weight gain and BMI score of the child (2). Lifelong supplementation of a high concentration of choline (5.0 g/kg choline chloride compared to 1.1 g/kg in the control diet group) significantly ameliorates Alzheimer’s disease pathology and its associated cognitive deficits (3). Insufficient uptake of choline is closely associated with fatty liver, muscle dysfunction and female reproductive related diseases (4, 5). For instance, circulating choline concentrations are decreased in patients with polycystic ovary syndrome (PCOS) (6). Taken together, this suggests that sufficient choline supply is important for growth performance and maintenance of health in both animals and humans.

However, excess choline uptake can induce thrombosis (7), cardiovascular disease (8) and stroke (9) as choline can be converted to trimethylamine (TMA) by the gut microbiome, absorbed into the blood and subsequently oxidized to generate trimethylamine-N-oxide (TMAO) by hepatic enzymes flavin-containing monooxygenase-3 (FMO3) (10). TMAO subsequently promotes the pathogenesis of the above diseases through activation of platelet hyper-reactivity, increase of foam cell formation and induction of inflammatory responses (11). The bioavailability of choline in mice is associated with the composition of the gut microbiome, especially TMA producing bacteria (12). A choline-rich, high-fat diet has been reported to alter gut microbial TMA production in mice which seems to be mediated by the alteration of colonic epithelial mitochondrial function (13). These findings suggest that there are close interactions and cross-talk between choline intake, gut microbiome and intestinal function that lead to different modes of catabolic pathway activation. Furthermore, in our previous study, we found that choline supplementation in the female pig increased ovulation rate, and altered CYP11A1 and LHR expression in ovarian tissue (14). A few studies also reported that gut microbiome composition is associated with ovarian development and can affect the pathogenesis of reproductive-related diseases (15, 16). However, if and how choline supplementation affects swine gut microbiome composition and intestinal function, which in turn affects ovarian development, is unknown. In this study, we aimed to assess if choline supplementation alters the gut microbiome and intestinal epithelial cell activity of gilts, resulting in changes to ovarian growth and development.

## 2. Materials and methods

### 2.1. Animal trial

Eighteen Yorkshire × Landrace gilts were recruited at 90 days of age and randomly divided into a Control group (Standard commercial corn and soybean meal-based diet, *n* = 9) and Choline group (control diet with additional 500 mg choline per kilogram of diet, *n* = 9), distributing littermates, body weight and backfat thickness between treatment groups. The diet ingredient composition and calculated nutrient contents are outlined in our previous publication which met estimated nutrient requirements for growing pigs [NRC (14, 17)]. Briefly, feed for grower phase contains 19.51% crude protein, 4.57% fat, and 2447 kcal/kg of net energy, while contains 17.16% crude protein, 3.84% fat and 2453 kcal/kg of net energy for finisher phase. An additional 500 mg/kg of choline was added to the feed of Choline group in both grower and finisher phases. This study was conducted at the Arkell Swine Research Station, University of Guelph, ON, Canada. All experimental procedures were approved by the University of Guelph’s Animal Care Committee (AUP #4068) and followed the Canadian Council of Animal Care Guidelines [CCAC (18)].

The animal trial design is described in our previous publication (14). In short, gilts were fed *ad libitum* with grower diet between 90 and 139 days of age and then switched to finisher diet between 140 and 186 days of age. During the grower stage, one of the gilts was culled due to lameness and 17 gilts successfully completed the trial. Individual gilts were weighed at the beginning and end of the trial, and overall bodyweight gain was calculated. The feed consumption of individual gilts was also recorded at the same time as bodyweight for calculation of the overall feed conversion ratio. Gilts were sacrificed at 186 days of age. Just prior to sacrifice, gilts were weighed and a 10 mL blood sample was collected from the orbital sinus. After collection of the whole blood in a 15 mL sterile centrifuge tube, blood was allowed to clot for 30 min at room temperature and the clot was removed by centrifuging at 3,000 × *g* for 10 min. Serum was transferred, aliquoted and stored at −80°C for later analysis. After sacrifice, the jejunum cross-section tissues (20 cm from the end of the stomach) were collected and kept in RNA later stabilization solution (Invitrogen, Cat#: AM7021, Carlsbad, CA, USA) and stored at −80°C for later total RNA extraction and gene expression analysis.

### 2.2. Hydrophilic interaction chromatography analysis of serum

As described in our previous publication (14), a solution composed of equal proportion of acetonitrile and methanol was added to the gilt serum to create an 80% organic solvent by mixing solution and serum in ratio of 4:1. The mixture was vortexed and passed through a 0.22 μm filter to remove particulates and protein. A sample of water treated the same way was conducted as a negative control. Samples were analyzed using Hydrophilic Interaction Chromatography (HILIC) in cooperation with the Department of Chemical Engineering and Applied Chemistry, University of Toronto, ON, Canada. The serum concentrations of TMA and TMAO were analyzed using MetaboAnalyst 4.0 developed by Chong el al. (19) (McGill University, QC, Canada).

### 2.3. Gut microbiome DNA extraction 16S rRNA sequencing

Feces were collected from the colon after slaughter at 186 days of age and were washed by adding Sodium Phosphate Buffer and centrifuged. Debris were collected and mixed with Sodium Phosphate Buffer and MT Buffer to lyse the cells. DNA was extracted from 400 mg feces using MP Biomedicals™ FastDNA™ SPIN DNA Isolation Kit for Feces (MP Biomedicals, Cat# 116570200, Santa Ana, CA, USA) according to the manufacturer’s directions. DNA quality and concentration was assessed using a NanoDrop 8000 (Thermo Fisher, Cat#: ND8000-GL, Waltham, MA, USA), gel electrophoresis and Qubit 2.0 (Invitrogen, CA, USA). High quality of microbiome DNA was then shipped to Novogene Biotech Co., Ltd., Beijing, China. for sequencing of the 16S rRNA V3–V4 hypervariable gene region.

A two-step PCR was using primers (341F CCTAYGGGRBGCASCAG; 806R GGACTACNNGGGTATCTAAT) with Illumina indices. Samples were diluted to equal concentrations and pooled into one library and sequenced on an Illumina MiSeq platform to generate 400–450 bp paired-end raw reads.

### 2.4. Bioinformatic analysis of gut microbiome data

The gut microbiome data processing and bioinformatic analysis approach was described by Cai et al. (20). Briefly, The Quantitative Insights into Microbial Ecology 2 (QIIME2) version 2020.11 pipeline was used for data cleaning, denoising and cluster analysis. SILVA database (132 release) was used as reference for taxonomic annotation of the amplicon sequence variant (ASV) using the QIIME2 feature-classifier classify-sklearn function. Furthermore, mitochondrion and chloroplast 16S rRNA genes were removed from all complete dataset by filter_pollution [taxa = c (“mitochondria,” “chloroplast”)] using R package microeco v0.12.0. The R-package phyloseq v1.22.3 was used to analyze the alpha and beta diversity of the gut microbiota in each group which includes rarefaction curve, observed taxa, Chao1 index, Shannon diversity. Finally, gut microbiome functional prediction was performed as previous described by Huyben et al. (21). Briefly, PICRUSt2 (v.2.3.0-b) and Kyoto Encyclopedia of Genes and Genomes (KEGG) database (22) were used to predict the functional profile, while Statistical Analysis of Taxonomic and Functional Profiles (STAMP) software version 2.1.3 was used to visualized the data. Significant differences for predictive metagenomic pathways were evaluated using Welch’s *t*-test.

### 2.5. CCK-8 assay

A porcine jejunal cell line, IPEC-J2 cells were used in all the *in vitro* assays. IPEC-J2 cells were cultured in DMEM/F12 media (WISENT, QC, Canada), supplemented with 10% fetal bovine serum (FBS) (WISENT, QC, Canada) and 1% penicillin-streptomycin (WISENT, QC, Canada) at 37°C with 5% CO_2_. Cells were digested using 0.25% trypsin-EDTA when they reached approximately 90% confluence, and were seeded to 96-well plate at a 5 × 10^4^ cells/mL density. After culturing for 24 h, cell culture media was discarded and rinsed two times with sterile PBS. Cells were refreshed with complete media containing 1.6 μM to 25 mM choline chloride, while another group only containing complete media served as a control. After treatment for 24 h, 10 μL of CCK-8 solution was added to each well, followed by incubation at 37°C for 2 h. The absorbance at 450 nm was measured using a Cytation 5 Cell Imaging Multi-Mode Reader (BioTek Instruments).

### 2.6. Caspase-3 activity assay in IPEC-J2 cells

IPEC-J2 cells were treated with 8, 40, and 200 μM choline chloride, respectively, for 24 h at 37°C in an incubator. The caspase-3 activity of the cells was determined as previously described (23) using a caspase-3 assay kit (Sigma-Aldrich, Oakville, ON, Canada). In short, after choline chloride treatment, IPEC-J2 cells were digested by using 0.25% trypsin-EDTA. The cell number of each well was counted using a hemocytometer after staining with 0.4% trypan blue. Cells were then lysed by using a lysis buffer provided by the kit, followed by a 4-h incubation on ice. Correlative reagents were added in accordance with the instruction of the kit. The caspase-3 activity was determined based on its ability to hydrolyze the acetyl-Asp-Glu-Val-Asp p-nitroanilide (Ac-DEVD-pNA) substrate into formazan p-nitroaniline (pNA) which appears yellow in color. The absorbance of pNA at 405 nm was measured by using a Cytation 5 Cell Imaging Multi-Mode Reader. The relative production of pNA was normalized to the total cell number.

### 2.7. Total RNA isolation and qRT-PCR

Total RNA from jejunum samples was isolated using Norgen Total RNA purification kit (Norgen Biotek, Cat#: 17200, Thorold, ON, CA) as previous described. Briefly, 10 mg of jejunal tissue from each gilt was thawed, homogenized in the provided lysis buffer, and total RNA was extracted per manufacturer’s directions. The concentration and 260/280 ratio of the collected total RNA was and assessed using a NanoDrop 8000 (Thermo Fisher, Cat#: ND8000-GL, Waltham, MA, USA) to ensure the samples were viable for reverse transcription. Total RNA sample was reverse transcribed into complementary DNA using iScript reverse transcription supermix kit (Bio-Rad, Cat#:1708841, Berkeley, CA, USA) according to manufacturer’s directions. Quantitative PCR was performed using Ssoadvanced™ Universal SYBR (Bio-Rad, Cat#:1725275, Berkeley, CA, USA). All the primer sets for gene expression were synthesized from Integrated DNA Technologies (IDT, Coralville, IA, USA). The primer sequence and expected product size are listed in Supplementary Table 1. For the gene expression normalization, GAPDH were used as reference genes. Relative quantification was conducted using the 2^–ΔΔCT^ method (24). The changes in gene transcription were presented as fold changes relative to the control group.

### 2.8. Statistical analysis

Statistical analysis was performed using SAS version 9.1 (SAS Institute, Cary, NC, USA) with each gilt as the experimental unit, dietary treatment as the fixed effect, and block as the random effect. A Shapiro-Wilk test was used to confirm a normal distribution of data. Statistical significance was measured with an independent two sample *t*-test. Differences at *P* < 0.05 were considered significant.

## 3. Results

### 3.1. Gilts growth performance

Our results showed that the overall bodyweight gain in the Choline group (108.3 ± 2.7) was slightly higher than that in the Control group (103.8 ± 3.5), and the feed to bodyweight conversion ratio in Choline group was lower than the control group (3.04 ± 0.09 vs. 3.29 ± 0.13, respectively). However, these differences were not found to be significantly different (Table 1).

**TABLE 1:** Overall grow performance of gilts supplemented with choline adding diet (mean ± SEM).

Item	Control	Choline	*P*-value
	***N* = 9**	***N* = 8**	
Overall bodyweight gain (kg)	103.8 ± 3.5	108.3 ± 2.7	0.335
Overall feed intake (kg)	339.2 ± 9.3	328.4 ± 12.2	0.488
Overall feed conversion ratio	3.29 ± 0.13	3.04 ± 0.09	0.158

### 3.2. Serum TMA and TMAO analysis

**FIGURE 1:**
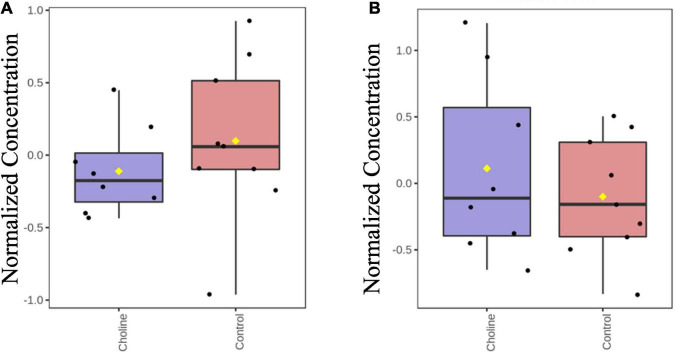
Boxplot of normalized concentrations of TMA **(A)** and TMAO **(B)** in serum of gilts at the age of day 186 in both Control and Choline diet group. *Y*-axis are represented as relative units. Values are presented by log transformed for *n* = 8 (Choline group) or 9 (Control group) gilts with medians indicated by horizontal lines within each box. Significance was measured using independent two sample *t*-test.

Hydrophilic Interaction Liquid Chromatography (HILIC) results showed that there were no differences in the concentration of TMA and TMAO in the serum of Control and Choline groups, suggesting that the supplemented concentration of choline is a safe dosage for gilt consumption (Figure 1).

### 3.3. Choline supplement altered gilts gut microbiome composition

**FIGURE 2:**
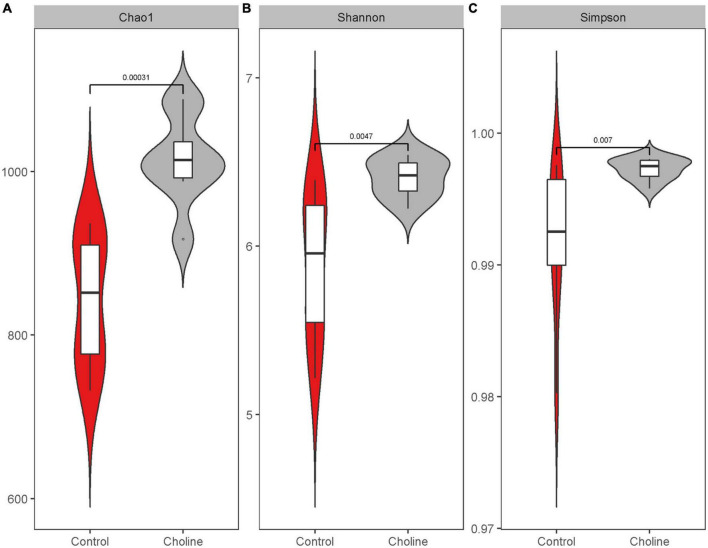
Boxplot of alpha diversity indices: Chao1 index **(A)**, Shannon index **(B)**, and Simpson index **(C)**. Alpha diversity indices are a representation of the microbiota species richness and evenness for a given number of individual samples, in which higher number means the microbiota is more diverse in the sequenced community. Samples were from the pig feces in 186 days of age feeding with control and choline diet. Data were average from 8 gilts in each group, significant difference was calculated using an independent two sample *t*-test.

We next characterized the dynamics of the swine gut microbiome in both Control and Choline supplemented group by analyzing the 16S rRNA V3–V4 region. In general, alpha-diversity increased when the gilts were fed with choline supplemented diet (Figure 2 and Supplementary Figure 1). A higher microbial richness, including the number of observed bacterial features and the Chao-1 index, were observed in the Choline group (Observed: Control 780.3 ± 127.4 vs. Choline 942.5 ± 127.6, *P* < 0.05; Chao-1: Control 820.5 ± 93.5 vs. Choline 987.8 ± 85.07, *P* < 0.05). Furthermore, the Shannon index, which has been widely used to assess the evenness of the community (25), was also significantly higher in the Choline group than in Control group (Control 5.9 ± 0.4 vs. Choline 6.4 ± 0.2, *P* < 0.05). These results suggest that choline supplementation enriched the diversity of gut microbiome in gilts. Moreover, the alpha-diversity of the gut microbiome was also found to be positively related to reproductive tract length and the number of antral follicle (*P* < 0.05) as shown in Supplementary Figure 2.

**FIGURE 3:**
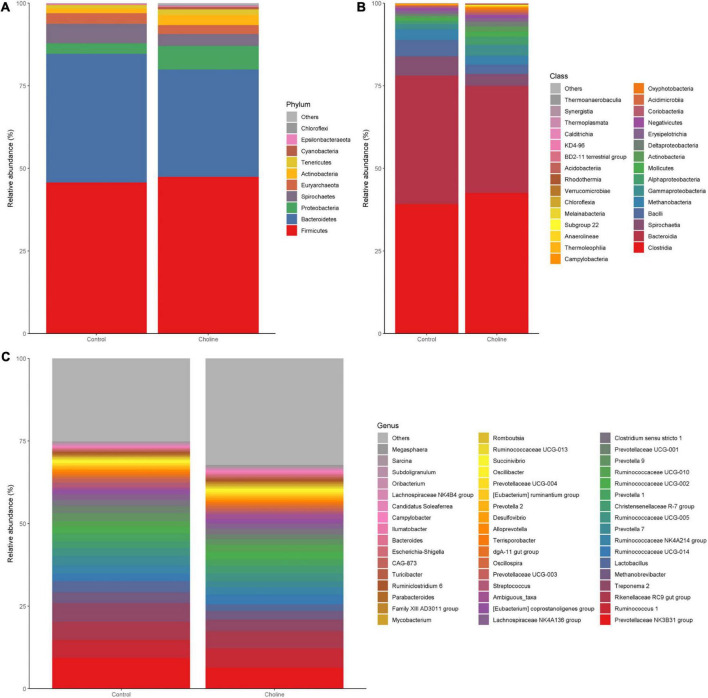
Bar plots of intestinal microbiota composition [**(A)** phylum level; **(B)** class level; **(C)** genus level] from feces of pigs feeding with Control and Choline diet for 3 months.

To study if choline supplementation in the diet affects gut microbiome relative abundance, beta-diversity of each group was analyzed and plotted by phylum (Figure 3A), class level (Figure 3B), and 50 of the most abundant bacteria in genus level (Figure 3C). Mean relative abundance at the phylum level in both the Control and Choline group were all dominated by *Firmicutes* (45.7% in Control group, 47.4% in Choline group), followed by *Bacteroidetes* (39% in Control group, 32.5% in Choline group). However, the subsequent abundant microbiota in the Control group were *Spirochaetes* (5.8%), *Euryarchaeota* (3.3%) and *Proteobacteria* (3.2%), while in the Choline group, the abundant microbiota groups were *Proteobacteria* (7.1%), *Actinobacteria* (3.1%), followed by *Euryarchaeota* (2.7%) (Figure 3A). At the class level, gut bacteria were dominated by *Clostridia*, *Bacteroidia*, *Bacilli*, *Methanobacteria*, *Spirochaetia*, *Gammaproteobacteria*, and *Alphaproteobacteria* in both groups. Among which, *Clostridia*, *Gammaproteobacteria*, and *Alphaproteobacteria* were more abundant in the Choline diet group than that of the Control group, while *Bacteroidia*, *Bacilli*, *Spirochaetia*, and *Methanobacteria* were found more enriched in the Control group than that in the Choline group (Figure 3B). At the genus level, *Prevotellaceae* NK3B31 group (9.3%), *Treponema* 2 (5.7%), *Rikenellaceae* RC9 gut group (5.6%), *Ruminococcus* 1 (5.5%), *Lactobacillus* (3.4%), and *Methanobrevibacter* (3.2%) dominated in the Control group, while *Prevotellaceae* NK3B31 group (6.4%), *Ruminococcus* 1 (5.9%), *Rikenellaceae* RC9 gut group (5.2%), *Treponema* 2 (3.4%), *Ruminococcaceae* UCG-014 (2.9%), and *Prevotella* 7 (2.9%) dominated the Choline diet group (Figure 3C). Furthermore, a correlation analysis between gut microbiota and the reproductive development indexes was conducted and 29 genera were observed to be significantly correlated with reproductive tract length, number of antral follicles, number of corpus luteum or the oviduct length (Supplementary Figure 3).

### 3.4. Metagenomic KEGG pathway prediction

**FIGURE 4:**
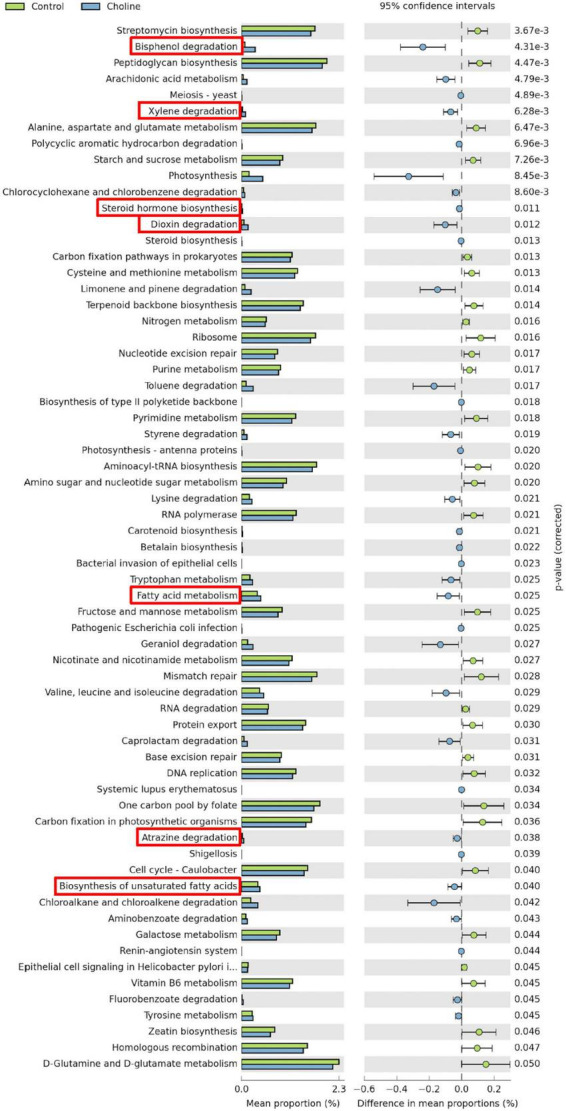
Statistical analysis of taxonomic and functional profiles (STAMP) plots of predictive metagenomic analysis using phylogenetic investigation of communities by reconstruction of unobserved states (PICRUSt) to infer function of the distal intestine microbiome (*N* = 8) of gilts after feeding 3 months of choline supplemented diet. Figures include all the significant different pathways generated from Kyoto Encyclopedia of Genes and Genomes (KEGG) Level 1–3. Pathways which are associated with reproductive health and ovarian development were highlighted in red boxes. *p*-values were generated by Welch’s *t*-test and then corrected with the false discovery rate (FDR). And *q* < 0.05 was considered significant.

Meta-genomic KEGG pathway prediction analysis showed that gut bacteria in the Choline diet group were mainly involved in bisphenol degradation, followed by toluene, chloroalkane and chloroalkene degradation. Among all the significant changed KEGG pathways, bisphenol degradation, xylene degradation, steroid hormone biosynthesis, dioxin degradation, fatty acid metabolism, atrazine degradation and biosynthesis of unsaturated fatty acids are related to reproductive health, which were significantly increased by the choline supplement diet (these pathways have been highlighted by red boxes in Figure 4).

### 3.5. Choline supplement changes jejunal gene expression in gilts

**FIGURE 5:**
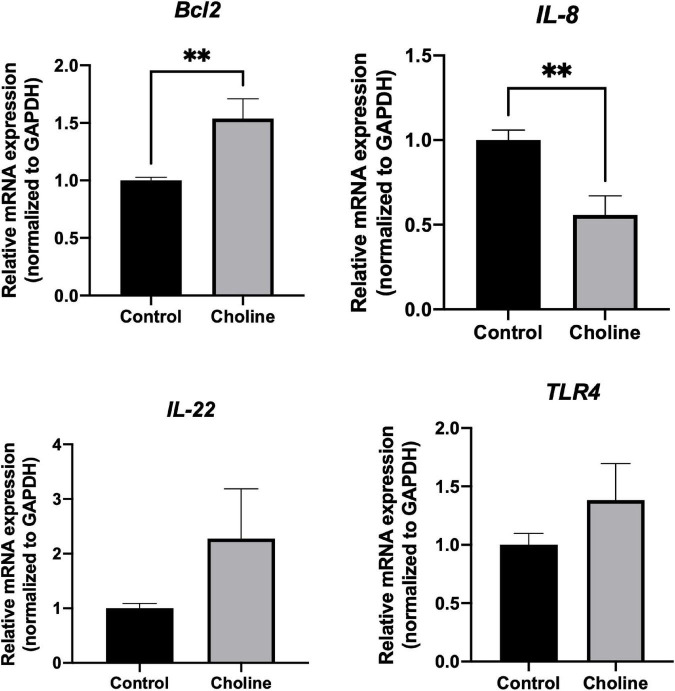
Relative mRNA expression of immune response-related genes including *Bcl2*, *IL-8*, *IL-22*, and *TLR4* in jejunum tissues (Normalized to *GAPDH*). Values are means ± standard error of *n* = 8 (Choline group) or 9 (Control group) gilts. Significance was measured using an independent two sample *t*-test. Astricts signify that the gene expression is significantly different in comparison to the control group (**P < 0.01).

To further study if choline supplementation alters the immune response related gene profile of the intestine, qPCR was performed to detect expression levels of some immune-responsive genes. There was no significant difference in the expression level of *IL-22* and *TLR-4* between the two groups (Figure 5). *Bcl2*, an anti-apoptotic gene, expression was significantly higher in Choline group compared to the Control group (*P* < 0.05), while the expression of *IL-8* was significantly lower in the Choline group compared to the Control group (*P* < 0.05). These results suggest that choline supplementation may also modulate intestine immune response.

### 3.6. Choline supplement affects IPEC-J2 proliferation and apoptosis *in vitro*

**FIGURE 6:**
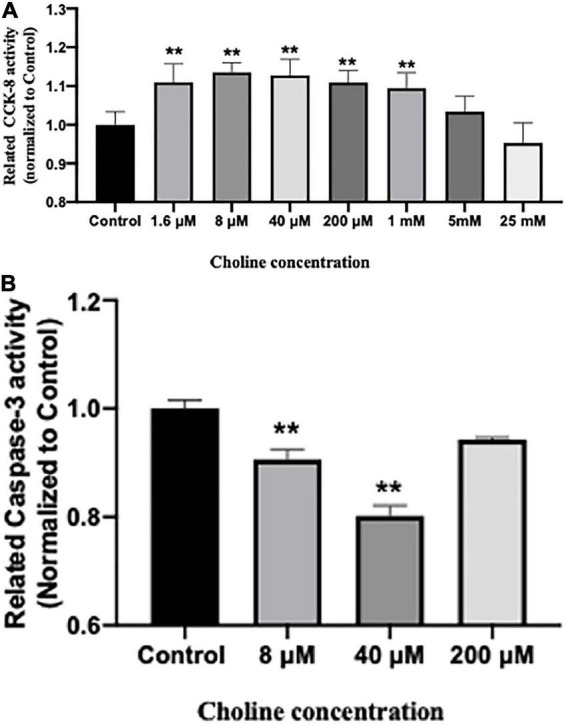
Choline chloride promotes proliferation **(A)** while inhibits apoptosis **(B)** of IPEC-J2 cells *in vitro*. Values are means ± standard error of three independent replicates. Significance was measured using a one-way ANOVA. Asterisks signify that the gene expression is significantly different in comparison to the control group (**P < 0.01).

Cell proliferative activity was tested using a CCK-8 kit after being treated with various concentrations of choline chloride for 24 h. The results showed that choline chloride significantly increased intestine cell proliferative activity in a concentration dependent manner starting at 1.6 μM (*P* < 0.05). No proliferation promoting effect was observed at choline concentrations greater than 5 mM (Figure 6A).

**FIGURE 7:**
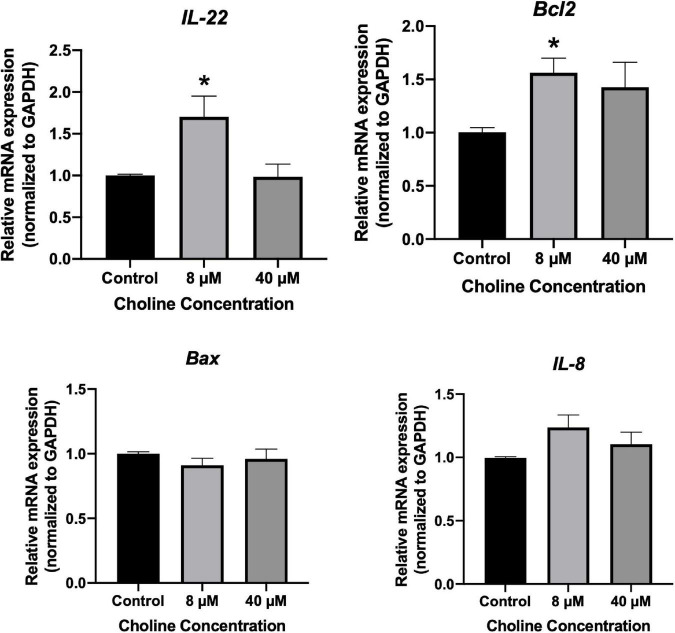
Relative mRNA expression of immune response-related genes in IPEC-J2 cell treated with 8 and 40 μM choline chloride for 24 h *in vitro*. Values are means ± standard error of three independent replicates. Significance was measured using a one-way ANOVA. Astricts signify that the gene expression is significantly different in comparison to the control group (*P < 0.05).

We further examined the influence of choline on intestine cell apoptosis using a Caspase-3 kit. The results showed that choline chloride significantly decreased the Caspase-3 activity at concentration of 8 and 40 μM (*P* < 0.05), while there was no inhibitory effect at 200 μM (Figure 6B). To test if choline chloride affects immune and apoptosis related gene expression *in vitro*, IPEC-J2 cells were treated with 8 and 40 μM of choline chloride for 24 h. As shown in Figure 7, choline chloride increased *IL-22* and *Bcl2* expression at the concentration of 8 μM (*P* < 0.05), but not 40 μM, while having no effect on Caspase-3, *IL-8* and *Bax* expression *in vitro*.

## 4. Discussion

As a main methyl donor, excessive choline supplementation is closely related to the development of altered DNA methylation related diseases, such as cancer and neurological disorders (26). This is mainly a result of the conversion of choline to TMA by the gut microbiota, which can further be catalyzed to TMAO in the liver (27, 28). In our study, after feeding the pigs with additional choline for 3 months, circulating TMA and TMAO levels showed no significant changes, suggesting that the supplemented dosage of choline in our study is within a safe range. Yoo et al. reported that plasma TMAO levels were only increased when choline was supplemented in a high-fat diet in mice, while it remained unchanged when a low-fat diet was supplemented with choline (13). In our trial, choline was mixed into the commercial standard diet that provides a fatty acid level meeting the minimum nutrient requirements. This was an important strategy to prevent the side effects caused by choline supplementation.

A clinical study showed that choline supplemented in cat food at fourfold greater than the basal diet significantly reduced cat bodyweight gain and body fat mass by reducing the food intake (29). Another study reported the same effect on bodyweight changes when supplementing a choline content 15-fold greater in comparison to the basal diet content in rats (30). Contrastingly, a choline deficient diet slowed the bodyweight gain of genetically modified obese mice (31). Taken together, this evidence suggests that choline intake is closely related to maintenance of body weight, possibly attributed to choline’s role in regulation of circulating glucose levels and fatty acid levels (31). In our study, overall feed intake was slightly decreased in the Choline group compared to the Control group which is consistent with previous findings (29). However, the overall bodyweight gain of gilts in our study was numerically increased when supplemented with choline, contrasting the results of previous studies outlined above. This difference could be attributed to the fact that the choline dosage supplemented in our study was only twofold greater than the basal concentration or attributed to the species differences between studies, especially cats which have the different metabolic priorities compared to pigs (32). However, our results highlight possible economic benefits for the pig industry as choline supplementation appeared to lower the overall feed conversion ratio.

We have previously reported that the corpus luteum number in each ovary in the Choline group (16.25 ± 2.88/ovary) was significantly higher in comparison to the Control group (5.56 ± 1.72/ovary), and choline also significantly increased the vagina-cervix-length of the gilts. Details can be found in our previous publication (14). Similar to our study, choline has also been shown to improve bovine oocyte quality and increased the ratio of embryo that developed to blastocyst after *in vitro* fertilization (33), which suggests that choline plays a role in reproductive tract development and female gamete production.

With the widespread use of antibiotics, an increasing number of human diseases have been associated with a decrease in gut microbiome diversity or microbiota dysbiosis (34). It was reported that lower microbiome diversity is closely associated with higher level of HOMA-IR, C-reactive protein in Twins UK cohorts (35) and higher risk of Colorectal Cancer (36), Crohn’s disease (37) and irritable bowel syndrome (IBS) (38). Moreover, a few female infertility-related diseases were also reported to be associated with a decrease in alpha diversity of the gut microbiome, such as PCOS (39) and endometriosis (40), suggesting the potential reproductive health benefits of gut microbiome diversity. Our data demonstrated that choline supplementation significantly increased the alpha-diversity of gut microbiome at the end of the trial, which were represented by higher Chao-1, Shannon, and Simpson indexes (Figure 2). This suggests that choline may help to shape a more stable, capable and resilient gut microbiome composition. However, a study conducted on weaned piglets found that supplementation of choline decreased the alpha-diversity index, including the ACE index and Shannon index (41). This difference may be explained by the fact that weaning is a unique period in a pig’s life that results in changes to the structure and function of the intestine due to the abrupt shift from a milk to plant-based solid feed diet (42). This could factor in to their different reactions to the choline supplemented diet.

It is reported that the gut microbiome influences estrogen activity in the host through the production of β-glucuronidase, an enzyme that reactivates estrogen by deconjugating estrogen with sex hormone binding globulin (SHBG) (43). Unbound estrogen is then able to bind to estrogen receptors and regulate its subsequent physiological downstream effects (43). Estrogen is critical for ovarian development and maintaining the normal menstrual cycle. However, excess active estrogen levels can lead to hormonal disorders and conditions including breast cancer, ovarian cancer, endometriosis and polycystic ovarian syndrome (44, 45). In our study, choline supplementation increased the abundance of a group of β-glucuronidase-producing bacteria including Eubacterium, *Alistipes*, *Ruminococcus*, and *Roseburia*, while decreasing the abundance of another group of β-glucuronidase producing bacteria such as *Bacteroides*, *Lactobacillus*, *Parabacteroides*, and *Prevotella*. This suggests that choline might influence ovarian development through maintaining the homeostasis of β-glucuronidase production *via* bacterial communities which in turn affects estrogen activity. This reasoning could also explain how choline supplementation affects ovarian follicular development, which has been demonstrated in our previous publication (14). This points to a complicated regulation axis among the gut microbiome, estrogen levels, and female reproductive development that exists to coordinate homeostasis in the body.

Using KEGG pathway prediction we found that the choline supplemented diet increased the abundance of bacteria that are mainly involved in the sex hormone biosynthesis and degradation of infertility-related environmental pollutants. Bisphenol A is an endocrine disruptor that exhibits high estrogenic activity and can bind to estrogen receptors, altering the downstream processes resulting in hormonal disorders (46). Xylene is an aromatic hydrocarbon that has been widely used in industry and medical technology as a solvent, but is reported to have a toxic effect on both the female reproductive system and fetuses in laboratory animals (47). Another environmental contaminant, Dioxin, has a long environmental half-life and readily accumulates in the food chain and body. Dioxin exposure has been reported to suppress rodent ovulation *in vivo* (48). Atrazine is a common herbicide with a long half-life in water and soil. It is an endocrine disruptor that can interrupt regular hormone secretion, leading to birth defects, ovarian tumors, and breast cancer (49, 50). By enriching gut bacteria that can degrade these kinds of dangerous environmental pollutants, such as *Pseudomonas* and *Sphingomonas*, choline supplementation may help to reduce the development of infertility or sub-fertility upon exposure.

The interaction among intestinal epithelial cells, immune system and gut microbiome is important for the immune response to pathogens and maintenance of overall health status. Increasing apoptosis of intestinal epithelial cells is associated with cell shedding and barrier loss (51), while proliferation of epithelial cells is required for the renewal of the intestinal epithelial layer and maintenance of absorptive and secretory functions (52). Data from our *in vitro* study showed that choline promotes intestinal epithelial cell proliferation, while inhibiting apoptosis by decreasing caspase-3 activity. Interestingly, Yan et al. also demonstrated that choline has the ability to attenuate olive oil lipid emulsion-induced enterocyte apoptosis, mainly through inhibition of the CELF1/AIF pathway *in vivo* and *in vitro* (53). However, in the present study, the specific pathway that choline activates in intestinal epithelial cells resulting in apoptosis, remains unclear and warrants future investigation.

Choline uptake is associated with the immune responsiveness of macrophages (54, 55) and hepatocytes (56). Diet supplemented with 1% choline influences bioenergetics of mitochondria in intestine epithelium which in turn alters the gut microbiome abundance and bacterial metabolomic functions (13). When we further examined the effect of choline supplementation on the immune response, we found that the expression of Bcl2, a protein that inhibits the action of pro-apoptotic protein such bax and bak, was decreased after both choline treatment *in vitro* and a choline supplemented diet *in vivo*. This also agrees with our result that choline decreases apoptotic activity *in vitro*. Additionally, a key mediator of inflammation, IL-8, was significantly lower in the intestinal tissue of the choline diet group compared to the control group. Several studies revealed that mice tend to develop gut inflammation when they have elevated apoptosis in the intestinal epithelium (57, 58). This may be mediated by the release of extracellular vesicles and various chemokines stimulated by the apoptotic signaling, which eventually activate and recruit immune cells (59). Detopoulou et al. found that higher intake of choline or betaine significantly decreased the circulating concentration of pro-inflammatory markers such as C-reactive protein and tumor necrosis-alpha (60), which further supports our finding.

IL-22 is a member of the IL-10 super family of cytokines in which it plays an important role in cell survival, wound healing and fighting pathogen invasion (61). Qi et al. demonstrated that serum IL-22 concentration was significantly decreased in patients with polycystic ovarian syndrome (PCOS), while administration of IL-22 improved PCOS phenotypes (16). In our study, IL-22 expression increased in intestine epithelial cells in a choline concentration dependent manner *in vitro*, while it did not show significantly different expression in the intestine with a choline supplemented diet *in vivo*. Interestingly, choline influences the gut’s bile acid metabolism by changing the diversity and composition of the gut microbiome (62). Bile acid is known to stimulate intestinal epithelial cells or immune cells to secrete IL-22 (16), which could circulate to the ovaries and in turn regulate ovarian development and function.

In conclusion, the findings from the current study reveal the beneficial effects of choline supplementation on gut microbiome composition and intestinal epithelial activity. It also offers insight into the potential mechanisms of how these changes may contribute to the “ovarian development promoting” effect of choline supplementation we have previously reported. We propose that there may be sophisticated cross-linkages among choline intake level, gut microbiome, epithelial function and the innate immune response, that in turn enhances a pathway for ovarian follicular development.

## Data availability statement

The data presented in this study are deposited in the NCBI SRA repository, accession number: PRJNA905186.

## Ethics statement

This animal study was reviewed and approved by the University of Guelph’s Animal Care Committee.

## Author contributions

XZ contributed to the experimental design, data collection and statistical analysis, and manuscript writing. LF performed the experimental preparation, animal care, and manuscript editing. DH and HC contributed to the bioinformatic analysis, figures generation, and manuscript editing. SD and NQ contributed to the animal care and sample collection. L-AH, BW, and JL contributed significantly to the experimental design, animal trial supervision, data statistical analysis, and manuscript editing and discussion. All authors contributed to the article and approved the submitted version.

## References

[1] Zeisel SH. Choline: critical role during fetal development and dietary requirements in adults. *Annu Rev Nutr.* (2006) 26:229–50. 10.1146/annurev.nutr.26.061505.111156 16848706 PMC2441939

[2] Moltó-Puigmartí C Obeid R Mommers M Eussen SJ Thijs C. Maternal plasma choline and betaine in late pregnancy and child growth up to age 8 years in the KOALA birth cohort study. *Am J Clin Nutr.* (2021) 114:1438–46. 10.1093/ajcn/nqab177 34113974 PMC8488875

[3] Velazquez R Ferreira E Knowles S Fux C Rodin A Winslow W Lifelong choline supplementation ameliorates Alzheimer’s disease pathology and associated cognitive deficits by attenuating microglia activation. *Aging Cell.* (2019) 18:e13037. 10.1111/acel.13037 31560162 PMC6826123

[4] Buchman AL. The addition of choline to parenteral nutrition. *Gastroenterology.* (2009) 137:S119–28. 10.1053/j.gastro.2009.08.010 19874943

[5] Du X Wu Z Xu Y Liu Y Liu W Wang T Increased Tim-3 expression alleviates liver injury by regulating macrophage activation in MCD-Induced NASH mice. *Cell Mol Immunol.* (2019) 16:878–86. 10.1038/s41423-018-0032-0 29735977 PMC6828758

[6] Troisi J Cinque C Giugliano L Symes S Richards S Adair D Metabolomic change due to combined treatment with myo-inositol, D-Chiro-Inositol and glucomannan in polycystic ovarian syndrome patients: a pilot study. *J Ovarian Res.* (2019) 12:25. 10.1186/s13048-019-0500-x 30904021 PMC6431025

[7] Zhu W Gregory JC Org E Buffa JA Gupta N Wang Z Gut microbial metabolite TMAO enhances platelet hyperreactivity and thrombosis risk. *Cell.* (2016) 165:111–24. 10.1016/j.cell.2016.02.011 26972052 PMC4862743

[8] Wang Z Klipfell E Bennett BJ Koeth R Levison BS Dugar B Gut flora metabolism of phosphatidylcholine promotes cardiovascular disease. *Nature.* (2011) 472:57–63. 10.1038/nature09922 21475195 PMC3086762

[9] Zhu W Romano KA Li L Buffa JA Sangwan N Prakash P Gut microbes impact stroke severity via the trimethylamine N-Oxide pathway. *Cell Host Microbe.* (2021) 29:1199–1208.e5. 10.1016/j.chom.2021.05.002 34139173 PMC8288076

[10] Liu Z-Y Tan X-Y Li Q-J Liao G-C Fang A-P Zhang D-M Trimethylamine N-Oxide, a gut microbiota-dependent metabolite of choline, is positively associated with the risk of primary liver cancer: a case-control study. *Nutr Metab.* (2018) 15:81. 10.1186/s12986-018-0319-2 30479648 PMC6245753

[11] Yang S Li X Yang F Zhao R Pan X Liang J Gut microbiota-dependent marker TMAO in promoting cardiovascular disease: inflammation mechanism, clinical prognostic, and potential as a therapeutic target. *Front Pharmacol.* (2019) 10:1360. 10.3389/fphar.2019.01360 31803054 PMC6877687

[12] Romano KA Vivas EI Amador-Noguez D Rey FE. Intestinal microbiota composition modulates choline bioavailability from diet and accumulation of the proatherogenic metabolite Trimethylamine-N-Oxide. *mBio.* (2015) 6:e02481-14. 10.1128/mBio.02481-14 25784704 PMC4453578

[13] Yoo W Zieba JK Foegeding NJ Torres TP Shelton CD Shealy NG High-Fat Diet–Induced colonocyte dysfunction escalates microbiota-derived trimethylamine N-oxide. *Science.* (2021) 373:813–8. 10.1126/science.aba3683 34385401 PMC8506909

[14] Zhan X Fletcher L Dingle S Baracuhy E Wang B Huber L-A Choline supplementation influences ovarian follicular development. *Front Biosci (Landmark Ed).* (2021) 26:1525–36. 10.52586/5046 34994167

[15] Gnainsky Y Zfanya N Elgart M Omri E Brandis A Mehlman T Systemic regulation of host energy and oogenesis by microbiome-derived mitochondrial coenzymes. *Cell Rep.* (2021) 34:108583. 10.1016/j.celrep.2020.108583 33406416

[16] Qi X Yun C Sun L Xia J Wu Q Wang Y Gut Microbiota-Bile acid-interleukin-22 axis orchestrates polycystic ovary syndrome. *Nat Med.* (2019) 25:1225–33. 10.1038/s41591-019-0509-0 31332392 PMC7376369

[17] National Research Council [NRC]. *Nutrient Requirements of Swine: Eleventh Revised Edition*. Washington, DC: The National Academies Press; (2012). 10.17226/13298

[18] CCAC. *Guidelines and Policies*. Available online at: https://ccac.ca/en/guidelines-and-policies/the-guidelines/

[19] Chong J Wishart DS Xia J. Using metaboanalyst 4.0 for comprehensive and integrative metabolomics data analysis. *Curr Protocols Bioinform.* (2019) 68:e86. 10.1002/cpbi.86 31756036

[20] Cai H Liao S Li J Liu Q Luo S Lv M Single and combined effects of clostridium butyricum and coccidiosis vaccine on growth performance and the intestinal microbiome of broiler chickens. *Front Microbiol.* (2022) 13:811428. 10.3389/fmicb.2022.811428 35547128 PMC9083122

[21] Huyben D Roehe BK Bekaert M Ruyter B Glencross B. Dietary lipid:protein ratio and n-3 long-chain polyunsaturated fatty acids alters the gut microbiome of atlantic salmon under hypoxic and normoxic conditions. *Front Microbiol.* (2020) 11:589898. 10.3389/fmicb.2020.589898 33424792 PMC7785582

[22] Kanehisa M Goto SK. EGG: kyoto encyclopedia of genes and genomes. *Nucleic Acids Res.* (2000) 28:27–30. 10.1093/nar/28.1.27 10592173 PMC102409

[23] Sudan S Zhan X Li JA. Novel probiotic bacillus subtilis strain confers cytoprotection to host pig intestinal epithelial cells during enterotoxic *Escherichia Coli* infection. *Microbiol Spectr.* (2022) 10:e01257-21. 10.1128/spectrum.01257-21 35736372 PMC9430607

[24] Livak KJ Schmittgen TD. Analysis of relative gene expression data using real-time quantitative PCR and the 2−ΔΔCT method. *Methods.* (2001) 25:402–8. 10.1006/meth.2001.1262 11846609

[25] Liu X Pan X Liu H Ma X. Gut microbial diversity in female patients with invasive mole and choriocarcinoma and its differences versus healthy controls. *Front Cell Infect Microbiol.* (2021) 11:704100. 10.3389/fcimb.2021.704100 34513727 PMC8428518

[26] Blusztajn JK Slack BE Mellott TJ. Neuroprotective actions of dietary choline. *Nutrients.* (2017) 9:815. 10.3390/nu9080815 28788094 PMC5579609

[27] Chan CWH Law BMH Waye MMY Chan JYW So WKW Chow KM. Trimethylamine-N-Oxide as one hypothetical link for the relationship between intestinal microbiota and cancer - where we are and where shall we go? *J Cancer.* (2019) 10:5874–82. 10.7150/jca.31737 31737123 PMC6843879

[28] Zhu W Zeneng W Wilson Tang WH Hazen SL. Gut Microbe-Generated TMAO from dietary choline is prothrombotic in subjects. *Circulation.* (2017) 135:1671–3. 10.1161/CIRCULATIONAHA.116.025338 28438808 PMC5460631

[29] Godfrey H. *Effects of Additional Dietary Choline on Food Intake, Body Weight and Composition, Respiratory Quotient, Serum Lipid Profile, and Serum Metabolic Signature in Post-Gonadectomy Kittens.* Guelph, Ont: University of Guelph (2021).

[30] Bagley BD Chang SC Ehresman DJ Eveland A Parker GA Peters JM Four-week dietary supplementation with 10- and/or 15-fold basal choline caused decreased body weight in Sprague Dawley rats. *Toxicol Ind Health*. (2017) 33:792–801. 10.1177/0748233717711361 28901218

[31] Wu G Zhang L Li T Lopaschuk G Vance DE Jacobs RL. Choline deficiency attenuates body weight gain and improves glucose tolerance in Ob/Ob mice. *J Obesity.* (2012) 2012:319172. 10.1155/2012/319172 22778916 PMC3385711

[32] Verbrugghe A Bakovic M. Peculiarities of one-carbon metabolism in the strict carnivorous cat and the role in feline hepatic lipidosis. *Nutrients.* (2013) 5:2811–35. 10.3390/nu5072811 23877091 PMC3739000

[33] Estrada-Cortés E Negrón-Peréz VM Tríbulo P Zenobi MG Staples CR Hansen PJ. Effects of choline on the phenotype of the cultured bovine preimplantation embryo. *J Dairy Sci.* (2020) 103:10784–96. 10.3168/jds.2020-18598 32896407

[34] Mosca A Leclerc M Hugot JP. Gut microbiota diversity and human diseases: should we reintroduce key predators in our ecosystem? *Front Microbiol.* (2016) 7:455. 10.3389/fmicb.2016.00455 27065999 PMC4815357

[35] Zouiouich S Loftfield E Huybrechts I Viallon V Louca P Vogtmann E Markers of metabolic health and gut microbiome diversity: findings from two population-based cohort studies. *Diabetologia.* (2021) 64:1749–59. 10.1007/s00125-021-05464-w 34110438 PMC8245388

[36] Ahn J Sinha R Pei Z Dominianni C Wu J Shi J Human gut microbiome and risk for colorectal cancer. *J Natl Cancer Institute.* (2013) 105:1907–11. 10.1093/jnci/djt300 24316595 PMC3866154

[37] Pascal V Pozuelo M Borruel N Casellas F Campos D Santiago A A microbial signature for Crohn’s disease. *Gut.* (2017) 66:813–22. 10.1136/gutjnl-2016-313235 28179361 PMC5531220

[38] Carroll IM Ringel-Kulka T Siddle JP Ringel Y. Alterations in composition and diversity of the intestinal microbiota in patients with diarrhea-predominant irritable bowel syndrome. *Neurogastroenterol Motility.* (2012) 24:521–30, e248. 10.1111/j.1365-2982.2012.01891.x 22339879 PMC3975596

[39] Thackray VG. Sex, microbes, and polycystic ovary syndrome. *Trends Endocrinol Metab.* (2019) 30:54–65. 10.1016/j.tem.2018.11.001 30503354 PMC6309599

[40] Ni Z Sun S Bi Y Ding J Cheng W Yu J Correlation of fecal metabolomics and gut microbiota in mice with endometriosis. *Am J Reproductive Immunol.* (2020) 84:e13307. 10.1111/aji.13307 32681566

[41] Qiu Y Liu S Hou L Li K Wang L Gao K Supplemental choline modulates growth performance and gut inflammation by altering the gut microbiota and lipid metabolism in weaned piglets. *J Nutr.* (2021) 151:20–9. 10.1093/jn/nxaa331 33245135

[42] Guevarra RB Lee JH Lee SH Seok M-J Kim DW Kang BN Piglet gut microbial shifts early in life: causes and effects. *J Animal Sci Biotechnol.* (2019) 10:1. 10.1186/s40104-018-0308-3 30651985 PMC6330741

[43] Baker JM Al-Nakkash L Herbst-Kralovetz MM. Estrogen-Gut microbiome axis: physiological and clinical implications. *Maturitas.* (2017) 103:45–53. 10.1016/j.maturitas.2017.06.025 28778332

[44] Sui Y Wu J Chen J. The role of gut microbial β-glucuronidase in estrogen reactivation and Breast cancer. *Front Cell Dev Biol.* (2021) 9:631552. 10.3389/fcell.2021.631552 34458248 PMC8388929

[45] Ervin SM Li H Lim L Roberts LR Liang X Mani S Gut Microbial β-Glucuronidases reactivate estrogens as components of the estrobolome that reactivate estrogens. *J Biol Chem.* (2019) 294:18586–99. 10.1074/jbc.RA119.010950 31636122 PMC6901331

[46] Eltoukhy A Jia Y Nahurira R Abo-Kadoum MA Khokhar I Wang J Biodegradation of endocrine disruptor bisphenol a by *Pseudomonas* putida strain YC-AE1 isolated from polluted soil, Guangdong, China. *BMC Microbiol.* (2020) 20:11. 10.1186/s12866-020-1699-9 31931706 PMC6958771

[47] Niaz K Bahadar H Maqbool F Abdollahi MA. Review of environmental and occupational exposure to xylene and its health concerns. *Excli J.* (2015) 14:1167–86. 10.17179/excli2015-623 26862322 PMC4743476

[48] Patel S Zhou C Rattan S Flaws JA. Effects of endocrine-disrupting chemicals on the ovary1. *Biol Reprod.* (2015) 93:1–9. 10.1095/biolreprod.115.130336 26063868 PMC6366440

[49] Sadeghnia H Shahba S Ebrahimzadeh-Bideskan A Mohammadi S Malvandi AM Mohammadipour A. Atrazine neural and reproductive toxicity. *Toxin Rev.* (2021) 41:1–14. 10.1080/15569543.2021.1966637

[50] Pathak RK Dikshit AK. Atrazine and human health. *IJE.* (2012) 1:14–23. 10.5923/j.ije.20110101.03 22499009

[51] Günther C Buchen B He G-W Hornef M Torow N Neumann H Caspase-8 controls the gut response to microbial challenges by Tnf-α-dependent and independent pathways. *Gut.* (2015) 64:601–10. 10.1136/gutjnl-2014-307226 25379949 PMC4392221

[52] Rees WD Tandun R Yau E Zachos NC Steiner TS. Regenerative intestinal stem cells induced by acute and chronic injury: the saving grace of the epithelium? *Front Cell Dev Biol.* (2020) 8:583919. 10.3389/fcell.2020.583919 33282867 PMC7688923

[53] Yan J Zhu J Gong Z Wen J Xiao Y Zhang T Supplementary choline attenuates olive oil lipid emulsion-induced enterocyte apoptosis through suppression of CELF1/AIF pathway. *J Cell Mol Med.* (2018) 22:1562–73. 10.1111/jcmm.13430 29105957 PMC5824412

[54] Sanchez-Lopez E Zhong Z Stubelius A Sweeney SR Booshehri LM Antonucci L Choline uptake and metabolism modulate macrophage IL-1β and IL-18 production. *Cell Metab.* (2019) 29:1350–1362.e7. 10.1016/j.cmet.2019.03.011 30982734 PMC6675591

[55] Snider SA Margison KD Ghorbani P LeBlond ND O’Dwyer C Nunes JRC Choline transport links macrophage phospholipid metabolism and inflammation. *J Biol Chem.* (2018) 293:11600–11. 10.1074/jbc.RA118.003180 29880645 PMC6065184

[56] Wolf MJ Adili A Piotrowitz K Abdullah Z Boege Y Stemmer K Metabolic activation of intrahepatic CD8+ T cells and NKT cells causes nonalcoholic steatohepatitis and liver cancer via cross-talk with hepatocytes. *Cancer Cell.* (2014) 26:549–64. 10.1016/j.ccell.2014.09.003 25314080

[57] Fuchs Y Steller H. Programmed cell death in animal development and disease. *Cell.* (2011) 147:742–58. 10.1016/j.cell.2011.10.033 22078876 PMC4511103

[58] El Andaloussi S Mäger I Breakefield XO Wood MJA. Extracellular vesicles: biology and emerging therapeutic opportunities. *Nat Rev Drug Discov.* (2013) 12:347–57. 10.1038/nrd3978 23584393

[59] Canbay A Feldstein AE Higuchi H Werneburg N Grambihler A Bronk SF Kupffer cell engulfment of apoptotic bodies stimulates death ligand and cytokine expression. *Hepatology.* (2003) 38:1188–98. 10.1053/jhep.2003.50472 14578857

[60] Detopoulou P Panagiotakos DB Antonopoulou S Pitsavos C Stefanadis C. Dietary choline and betaine intakes in relation to concentrations of inflammatory markers in healthy adults: the ATTICA study. *Am J Clin Nutr.* (2008) 87:424–30. 10.1093/ajcn/87.2.424 18258634

[61] Dudakov JA Hanash AM van den Brink MRM. Interleukin-22: immunobiology and pathology. *Annu Rev Immunol.* (2015) 33:747–85. 25706098 10.1146/annurev-immunol-032414-112123 PMC4407497

[62] Suga T Yamaguchi H Ogura J Shoji S Maekawa M Mano N. Altered bile acid composition and disposition in a mouse model of non-alcoholic steatohepatitis. *Toxicol Appl Pharmacol.* (2019) 379:114664. 10.1016/j.taap.2019.114664 31306673

