# Supplementary material for: Choline supplementation regulates gut microbiome diversity, gut epithelial activity, and the cytokine gene expression in gilts
Source: Front Nutr. 2023 Feb 2;10:1101519. doi: 10.3389/fnut.2023.1101519 (PMC9931747; doi:10.3389/fnut.2023.1101519)
Supplement: Supplementary file 1 [file Data_Sheet_1.pdf]

Supplementary Figure 1

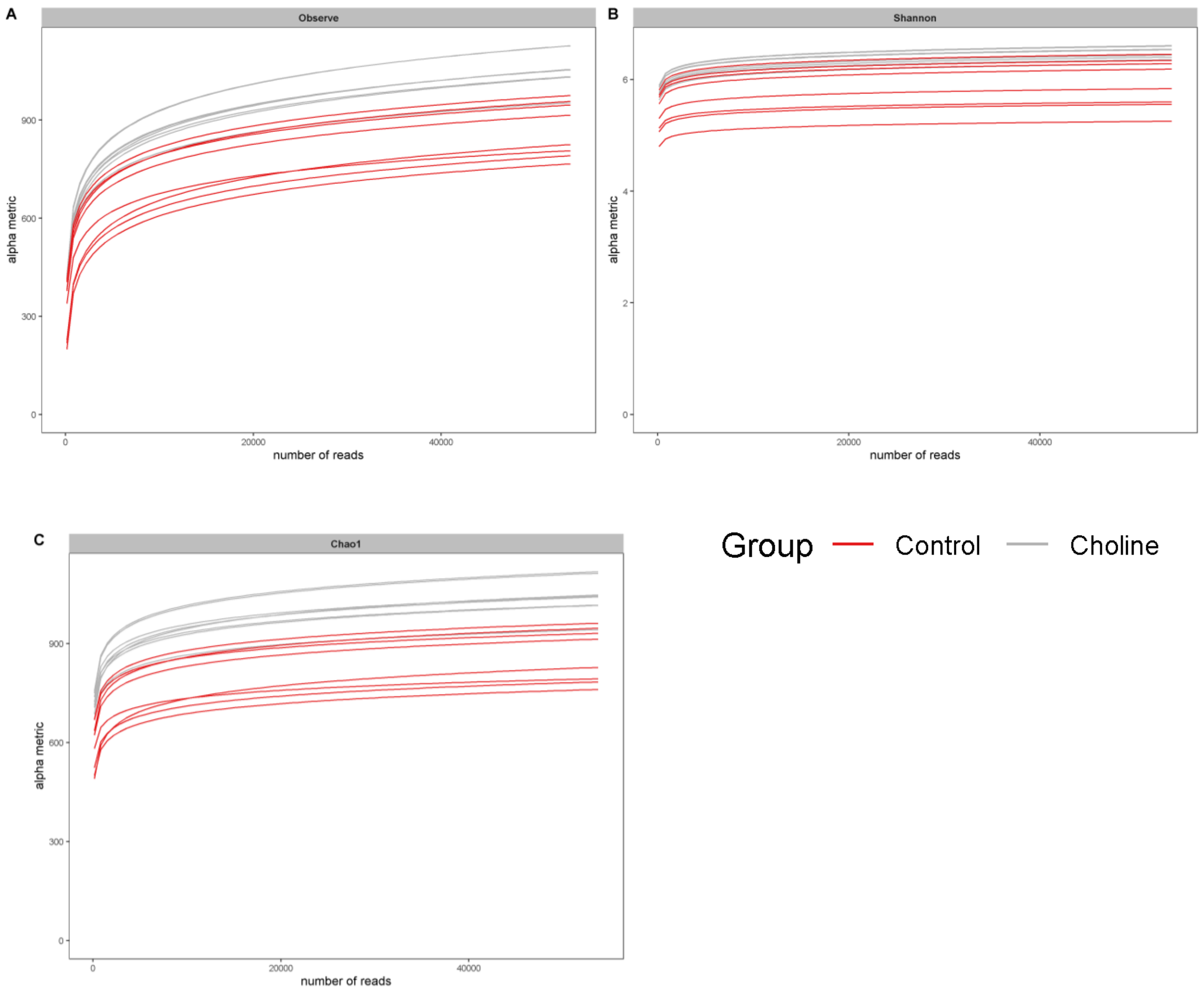

Figure S1. Rarefaction curves of Observe OTUs (A), Shannon index (B) and Chao1 index (C). Samples were from the pig feces in 186 days of age feeding with control and choline diet. Samples from Control group were represented in red color, while Samples from Choline group were represented in grey color.

Supplementary Figure 2

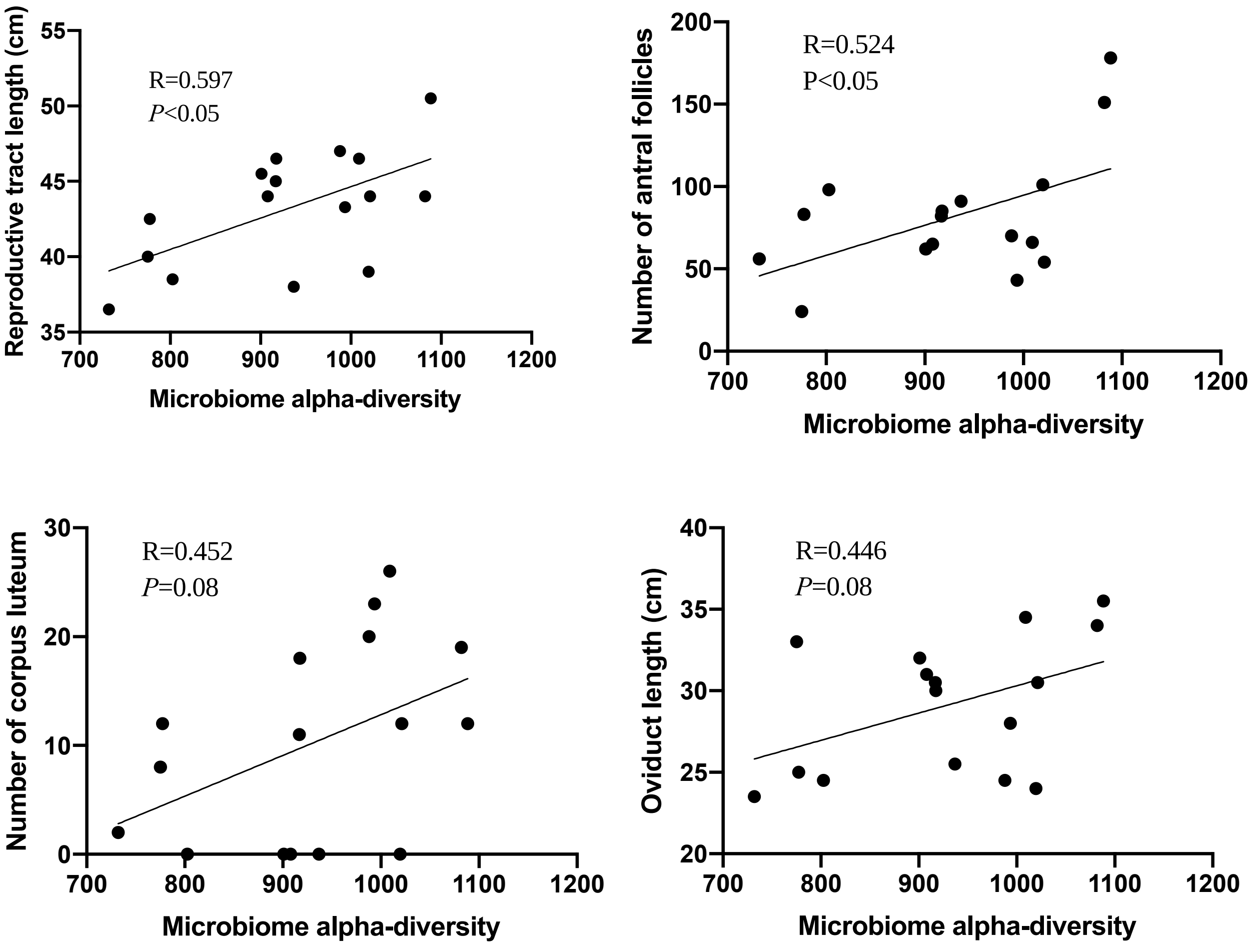

Figure S2. Scatter plot with correlation coefficient of gut microbiome alpha-diversity and reproductive development indices(n=16).

Supplementary Figure 3

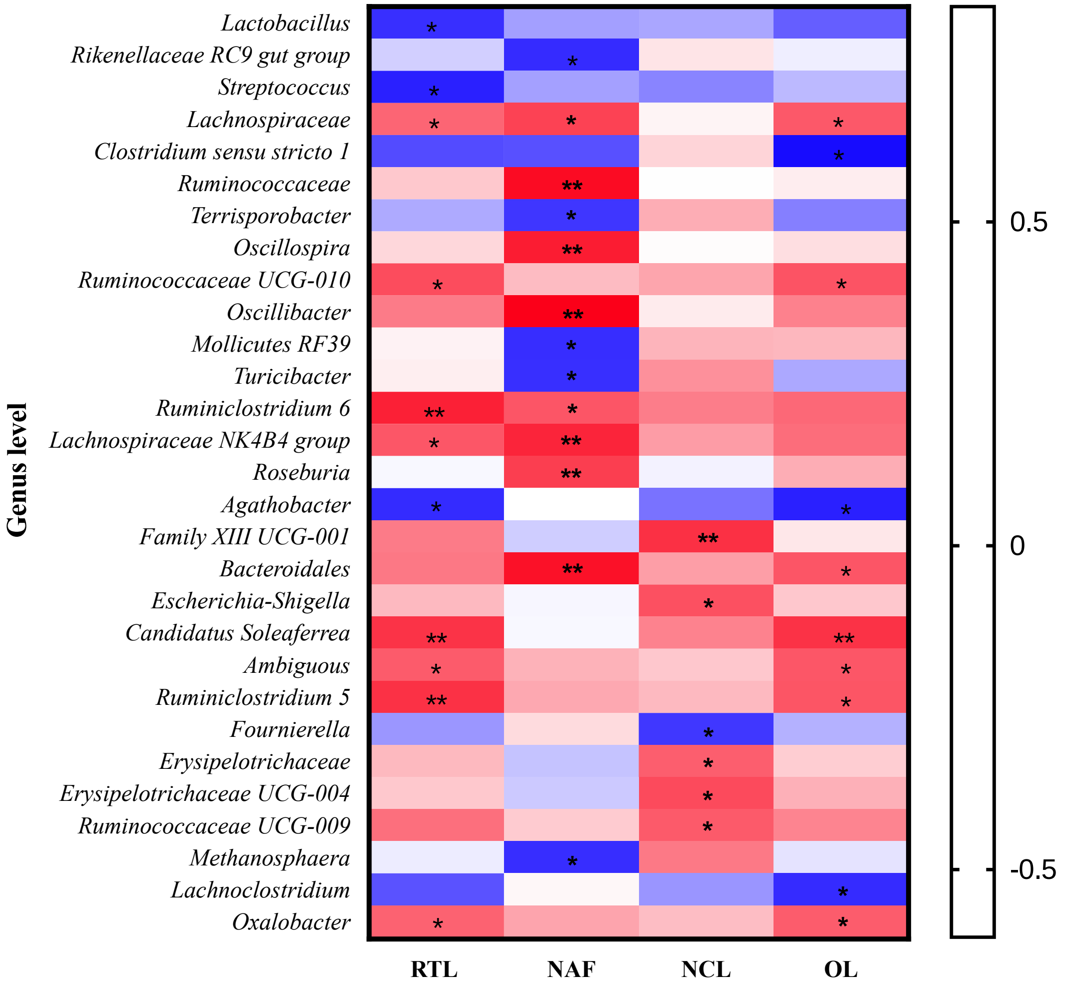

Figure S3. Heat map of Pearson correlation analysis between the the gut microbiome in genus level and the reproductive development indices (n=16). RTL: reproductive tract length; NAF: number of antral follicles; NCL: number of corpus Luteum; OL: oviduct length. $*P < 0.05$, $**P < 0.01$

Supplementary Table 1

Table S1. Primers for gene expression detection

Gene	Product size	Forward 5’-3’	Reverse 5’-3’	Annealing Tm	Accession number
GAPDH	147	TCGGAGTGAACGGATTTGGC	TGCCGTGGGTGGAATCATAC	60°C	NM_001206359.1
Bcl2	147	GGATAACGGAGGCTGGGATG	TTATGGCCCAGATAGGCACC	58°C	XM_021099593.1
IL-8	62	GCTCTCTGTGAGGCTGCAGTT	TTTATGCACTGGCATCGAAGTT	60°C	NM_213867.1
IL-22	112	GATGAGAGAGCGCTGCTACCTGG	GAAGGACGCCACCTCCTGCATG T	60°C	XM_021091967.1
Bax	153	ATGATCGCAGCCGTGGACACG	ACGAAGATGGTCACCGTCTGC	59°C	XM_003127290.5

GAPDH, glyceraldehyde-3-phosphate dehydrogenase; *Bcl2*, B-cell lymphoma 2; *IL-8*, Interleukin 8; *IL-22*, Interleukin-22; *Bax*, BCL2 Associated X
